# Novel QSAR Models for Molecular Initiating Event Modeling in Two Intersecting Adverse Outcome Pathways Based Pulmonary Fibrosis Prediction for Biocidal Mixtures

**DOI:** 10.3390/toxics9030059

**Published:** 2021-03-16

**Authors:** Myungwon Seo, Chong Hak Chae, Yuno Lee, Ha Ryong Kim, Jongwoon Kim

**Affiliations:** 1 Chemical Safety Research Center, Korea Research Institute of Chemical Technology, Daejeon 34114, Korea; mwseo@krict.re.kr; 2 Data Convergence Drug Research Center, Korea Research Institute of Chemical Technology, Daejeon 34114, Korea; chchae@krict.re.kr; 3 Drug Information Platform Center, Korea Research Institute of Chemical Technology, Daejeon 34114, Korea; yunolee@krict.re.kr; 4 College of Pharmacy, Daegu Catholic University, Gyeongsan 38430, Korea

**Keywords:** adverse outcome pathway network, molecular initiating event, biocide, mixture toxicity, pulmonary fibrosis, quantitative structure–activity relationship

## Abstract

The adverse outcome pathway (AOP) was introduced as an alternative method to avoid unnecessary animal tests. Under the AOP framework, an in silico methods, molecular initiating event (MIE) modeling is used based on the ligand-receptor interaction. Recently, the intersecting AOPs (AOP 347), including two MIEs, namely peroxisome proliferator-activated receptor-gamma (PPAR-γ) and toll-like receptor 4 (TLR4), associated with pulmonary fibrosis was proposed. Based on the AOP 347, this study developed two novel quantitative structure-activity relationship (QSAR) models for the two MIEs. The prediction performances of different MIE modeling methods (e.g., molecular dynamics, pharmacophore model, and QSAR) were compared and validated with in vitro test data. Results showed that the QSAR method had high accuracy compared with other modeling methods, and the QSAR method is suitable for the MIE modeling in the AOP 347. Therefore, the two QSAR models based on the AOP 347 can be powerful models to screen biocidal mixture related to pulmonary fibrosis.

## 1. Introduction

Biocides are used extensively industrially, professionally, and personally to control harmful organisms. Biocides contain active substances and co-formulants, such as solvent, stabilizer, and wetting agent [1]. According to the European Union’s (EU) Biocidal Products Regulation (BPR) [2] and South Korea’s “Household Chemical Products and Biocides Safety Act (or K-BPR) [3]” for biocide regulation, the toxicological effects in mixture of chemicals, namely additivity, synergism, and antagonism, of biocidal products should be considered when approving a biocide. In the case of a single compound’s toxicological effect, methods of QSAR or quantitative structure-property relationship (QSPR) were considered to assess the risk of a single compound such as a pesticide in the EU [4,5]. However, mixture toxicity effects occur when the mixture components interact. Unless there is explicit evidence of synergistic toxicity, an additive toxicity approach, which assumes the concentration addition concept as a default, can be employed under the regulations. However, data on synergistic toxicity effects among mixture components are lacking [6,7]. In addition, conducting toxicity tests for all mixtures conventionally can be an economic burden to the chemical industry and unfeasible because the number of conceivable mixture combinations is extremely large.

The adverse outcome pathway (AOP) has been presented as an alternative approach to in vivo testing by integrating in silico and in vitro methods [8]. The AOP framework means that a logical sequence of a biological system, from the toxic chemical to the production of toxic effect, consists of the molecular initiating event (MIE), adverse outcome (AO), key event (KE), and the key event relationship (KER). The MIE describes the interaction between the chemical and the target protein, and the AO implies the occurrence of adverse effects at the level of biological organization. The KE is an intermediate key event that connects the MIE and AO, and KER describes the linkage between the events [9]. Because a single AOP may not be sufficient in interpreting all events of a biological system, the AOP network, which shares at least one component of related AOPs, was suggested [10]. The AOP network provides information on AOP interactions and potential chemical effects in the biological system for toxicity risk assessment. MIE modeling in AOP network research has been applied for the prediction of the final biological activity related to the interaction between a target chemical and a receptor [11,12,13]. In silico methods include molecular dynamics (MD), pharmacophore models, and quantitative structure–activity relationships (QSARs). MD is a simulation method adopted to analyze the physical movements of molecules on a time-based atomic scale. The pharmacophore model explores and uses the pattern of common features correlated with the biological activity of a molecule or biological target. The QSAR model is based on regression or classification models and finds a correlation between the molecular structure and biological activity.

Pulmonary fibrosis is a respiratory disease involving scar tissue accumulation. The pulmonary fibrosis mechanism was identified as the inactivation of peroxisome proliferator-activated receptor-gamma (PPAR-γ) and was referred to as the AOP 206 [14,15]. However, the AOP 206, having one MIE of PPAR-γ only, has a limitation to consider the synergistic interaction for mixture toxicity of compounds using the MIE modeling based on the in silico method. The two intersecting AOPs (AOP 347) for pulmonary fibrosis, the respiratory disease was recently proposed [16]. The AOP 347 suggests that the combination of two different MIEs, including the activation of toll-like receptor 4 (TLR4) as well as the inactivation of PPAR-γ, promotes pulmonary fibrosis as a cocktail effect. The AOP 347 consists of two MIEs, six KEs, and one AO (for details, see [16]).

In previous studies, QSAR models for one PPAR-γ and two TLR4 activations were developed to predict agonist compounds [17,18,19]. However, these QSAR models were mainly developed using the drug compounds as training data for predicting drug candidates. Because the QSAR models’ training data did not consider biocides’ physicochemical properties and PPAR-γ activation was not contained in the AOP 347, it has a limitation for predicting pulmonary fibrosis of biocides using the previous QSAR models.

Therefore, the objectives of this study were (i) to develop QSAR models for MIE modeling in the AOP 347-based pulmonary fibrosis prediction for binary biocidal combination, (ii) to compare and validate the prediction performance of different MIE modeling approaches (i.e., MD, pharmacophore model, and QSAR) with in vitro test data of human lung cell; and (iii) to suggest two QSAR models to predict binary chemicals associated with pulmonary fibrosis. For MIE modeling, the inactivation of PPAR-γ for target biocides was predicted using MD and QSAR modeling. For predicting the activation of TLR4 as the toll-like receptor 4/myeloid differentiation factor 2 (TLR4/MD2) complex protein, pharmacophore, and QSAR modeling were conducted for target biocides. In this study, five compounds of PPAR-γ inactivation and five compounds TLR4 activation were selected from compounds conflictingly predicted using two different MIE modeling. Ten compounds were used for the in vitro testing of human lung cells for evaluating MIE modeling methods.

## 2. Materials and Methods

**Figure 1:**
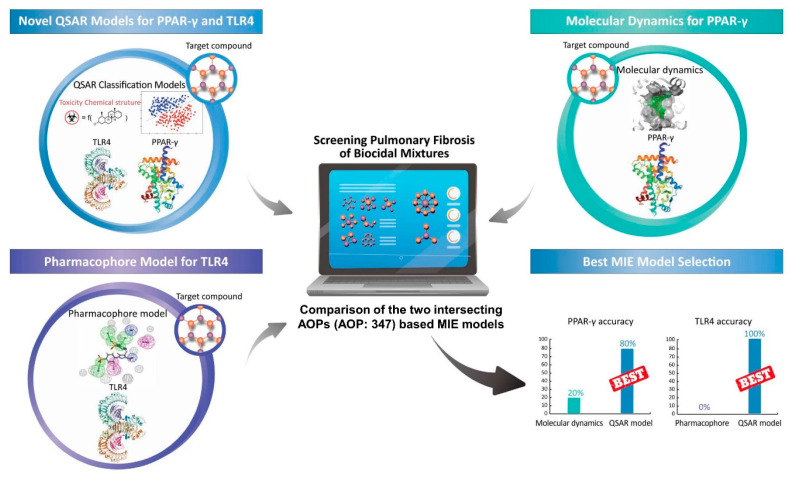
The scheme of the MIE modeling approach in the two intersecting AOPs (AOP 347).

The MIE modeling approach of two intersecting AOPs (AOP 347) involves three modeling methods, including MD, pharmacophore model, and a quantitative structure-activity relationship (QSAR) model (Figure 1). The best MIE modeling approach was selected by comparing outcomes of in vitro experiments.

### 2.1. Data Sets

#### 2.1.1. Co-Exposure Biocide Data Collection

Our previous meta-study [20], investigated real combined inhalation exposures to biocides including, 1733 real products containing two or more biocides marketed in South Korea. Of the 1733 products, except 130 duplicated combinations, we confirmed 768 combined exposure scenarios. A total of 314 biocides with a high frequency of co-exposure patterns were identified. We searched the structures of the 314 biocides based on the chemical abstracts service (CAS) number and downloaded 255 biocide structures from the PubChem database (https://pubchem.ncbi.nlm.nih.gov/, accessed on 8 December 2020) (Supplementary File S1) [21]. Because 59 biocides did not include structural information in the PubChem database, they were excluded from the MIE modeling.

#### 2.1.2. Receptor and Ligand Data Collection for MD and Pharmacophore Modeling

The structure of the four reference antagonists of PPAR-γ, namely betulinic acid [22], SR1664 [23], GW9662 [24], and FH535 [25], were downloaded from the PubChem database [21]. The crystal structure of PPAR-γ (PDB code: 6C5T) was retrieved from the Protein Data Bank (PDB) and used as the receptor structure for MD modeling [26]. The crystal structure of human TLR4 with LPS-bound MD-2 complex (PDB code: 3FXI) was used to extract the pharmacophore features of the TLR4-MD2 activator [27].

#### 2.1.3. Data Collection for QSAR Modeling

To develop the QSAR models, we searched for bioassay data of PPAR-γ (Uniprot ID: P37231) and TLR4 (Uniprot ID: O00206) in PubChem. We downloaded the bioassay data of PPAR-γ and TLR4 and filtered it using the criteria that data include biological activity nearly half-maximal inhibitory concentration (IC50) and the half-maximal effective concentration (EC50), respectively. In each filtered dataset, compound structures of PPAR-γ and TLR4 were downloaded from the PubChem database based on the CID number and duplicated structures were removed. We collected 3084 compounds for PPAR-γ inactivation, comprising 903 positive datasets and 2181 negative datasets, and 118 compounds for TLR4 activation comprising 60 positive datasets and 58 negative datasets. The dataset of QSAR models was collected by focusing on predicting MIEs such as PPAR-γ inactivation, TLR4 activation. In the PPAR-γ inactivation, the positive dataset contained antagonists, and the negative dataset included agonists and non-binders. In the TLR4 activation, the positive dataset contained agonists, and the negative dataset was grouped into antagonists and non-binders.

#### 2.1.4. Selection of Test Substance

We performed an in vitro experiment to evaluate the MIE modeling approach and compare the in silico methods of MIE modeling. Among the compounds predicted using the MIE modeling methods, test substances were selected based on the criteria that (i) the substance is experimentally available and (ii) the substance includes an opposite predicted result compared between MD or pharmacophore modeling and QSAR modeling. Moreover, the structural diversity of tested substances was considered. The test substances are listed in Table 1. Stock solutions of test substances were prepared and diluted in distilled water (DW) or DMSO.

### 2.2. MD Modeling for PPAR-γ

#### 2.2.1. Data Preparation

The Schrodinger Molecular Modeling Suite 2020-1 was used for data preparation for MD. Biocides and PPAR-γ antagonists were prepared using the LigPrep 5.3 module with default parameters. The structural defects correction and energy minimization of PPAR-γ and TLR4 was performed in the Protein Preparation Wizard module [28].

#### 2.2.2. Calculation of MD and MM-GBSA

In the crystal structure of PPAR-γ, the receptor grid was centered on the bound ligand of the crystal structure, and the box size was set to 20 Å by default. The standard precision mode flexible docking protocol was used for molecular docking calculation with default parameters in the Glide 8.6 module [29]. The best-docked protein-ligand complex with the lowest GlideScore and good binding poses without bad contact was eye-selected for subsequent MD simulation.

For each protein-ligand complex, the binding free energy was re-evaluated through MD simulation with end-point molecular mechanics-generalized born surface area (MM-GBSA) computation. The SystemBuilder module was used for solvation with predefined SPC water molecules in an orthorhombic box with a dimension of 10 Å spaces with all other parameters set to default. A 10 ns equilibrium MD trajectory was generated for each structure in an explicit solvent model and NPT ensemble condition using the Desmond 6.1 module [30].

Structural snapshots were saved every 50 ps during the final 1 ns MD simulation and used for MM-GBSA binding free energy calculation. The OPLS3e force field, VSGB 2.0 solvation model, and default prime parameters in the Prime 5.9 and MM-GBSA 3.0 modules were used for the MM-GBSA calculations [31,32].

### 2.3. Pharmacophore Modeling for TLR4

The Receptor–Ligand Pharmacophore Generation module implemented in Discovery Studio 2018 software was applied to obtain the pharmacophore model considering the complementary interaction for binding of lipopolysaccharide (LPS) [33]. The model can be used as a 3D query for retrieving molecules that activate the interaction between TLR4 and MD-2 instead of LPS. Based on the highest selectivity predicted by the genetic function approximation model, the best pharmacophore model was selected.

### 2.4. QSAR Modeling for PPAR-γ and TLR4

#### 2.4.1. Data Curation

The collected dataset of PPAR-γ inactivation and TLR4 activation from the PubChem contains synthetic compounds associated with drug development. Because most biocides are small, the synthetic and biocide compounds do not partially overlap in the chemical space of the physicochemical features. Thus, to develop a QSAR model for predicting biocide activity, the collected dataset should be filtered to the compound associated with biocide structure.

Principal component analysis (PCA) was performed to extract compound data correlating with the biocide from the collected data. We calculated the compound’s physicochemical properties to calculate the PCA score and used the calculated 2622 physicochemical properties as molecular descriptors. Chemical space was constructed using PCA scores and the average biocide similarity distance in chemical space was calculated based on Euclidean distance. Next, if the distance between biocide and collected data was below the average biocide similarity distance, that data was considered modeling data. To solve data imbalance, undersampling was performed using the Tomek link (T-link) method [34].

#### 2.4.2. Calculation of Molecular Descriptors

We used alvaDesc to calculate molecular descriptors [35]. The alvaDesc software can be used to calculate various molecular descriptors and molecular fingerprints. This tool calculates more than 5300 descriptors belonging to 33 classes, such as molecular property, topological, and pharmacophore. The molecular descriptors of all compounds were calculated, and variable reduction was performed by removing at least one missing value or all missing values from the results of molecular descriptor calculation.

#### 2.4.3. Model Development and Validation

In order to develop two classification-based QSAR models of PPAR-γ inactivation and TLR4 activation, the support vector machine (SVM) algorithm was used for model development. The SVM algorithm parameters used were LibSVM, RBF kernel, C value, and gamma. C value and gamma were applied 5.0 and 1.0 for model training. The parameters were selected based on model performance. The optimized descriptors of the models were selected by using the genetic algorithm (GA) [36]. We randomly divided the model training dataset and external validation dataset at a ratio of 8:2. We performed internal validation of a QSAR model by using 10-fold cross-validation and optimized the parameters of SVM. Model evaluation was performed five times with five external validation datasets.

The model’s performance was evaluated as accuracy (ACC), the area under the curve (AUC), sensitivity, specificity, balance accuracy (BA), and Matthews correlation coefficient (MCC) using the external validation dataset. For model development, we used RapidMiner Studio 9.7 [37].

#### 2.4.4. Y-Randomization

The Y-randomization test is used to evaluate the robustness of the model [38]. During Y-randomization, Y-values (end-point values) of the training data were randomly shuffled, and random models were built based on the shuffled training data repeatedly. *ACC* and *MCC* [39] were calculated in both original and random models and z-scores were calculated as follows,

(1) $$Z_{ACC}=\;\frac{ACC_{ori}-\;ACC_{ran}^{avg}}{\sigma_{ran}^{ACC}}$$

(2) $$Z_{MCC}=\;\frac{MCC_{ori}-\;MCC_{ran}^{avg}}{\sigma_{ran}^{MCC}}$$

where $Z_{ACC}$ and $Z_{MCC}$ represent the z-scores of the *ACC* and *MCC* of the models, respectively. $ACC_{ori}$ and $MCC_{ori}$ represent *ACC* and *MCC* values of the original model, trained using the correct training data set, respectively. $ACC_{ran}^{avg}$ or $MCC_{ran}^{avg}$ and $\sigma_{ran}^{ACC}$ or $\sigma_{ran}^{MCC}$ are the averages and standard deviations of the *ACC* and *MCC* values of random models, respectively. If the model’s original data is robust compared with the model of random data, the z-score is higher than 3.0.

### 2.5. In Vitro Study

#### 2.5.1. Cell Culture

A549 human lung adenocarcinoma cells were obtained from the Korean Cell Line Bank (Seoul, Korea) and cultured in RPMI supplemented with 5% fetal bovine serum (Biotechnics Research Inc., Lake Forest, CA, USA), penicillin (100 units/mL), and streptomycin (100 μg/mL) at 37 °C and 5% CO_2_, under saturating humidity. HEK-Blue-hTLR4 cells were purchased from InvivoGen (San Diego, CA, USA) and cultured in DMEM supplemented with 10% fetal bovine serum (Biotechnics Research Inc., Lake Forest, CA, USA), penicillin (100 units/mL), and streptomycin (100 μg/mL) at 37 °C and 5% CO_2_, under saturating humidity.

#### 2.5.2. Western Blot Analysis for Evaluating PPAR-γ Inactivation

A549 cells were seeded in 6-well plates at 6 × 10^4^ cells/well, incubated for 24 h at 37 °C, and treated with the test substances. After the indicated time periods, the cells were washed twice with phosphate-buffered saline and lysed using the radioimmunoprecipitation assay buffer (Thermo Scientific, Waltham, MA, USA) containing the protease inhibitor cocktail (GenDEPOT, Barker, TX, USA), phosphatase inhibitor (BioVision, Milpitas, CA, USA), and 0.1% SDS. Equal amounts of proteins were denatured in Laemmli sample buffer (Bio-Rad, Hercules, CA, USA), and denatured protein (15 μg) was loaded onto a 10% acrylamide gel. Following separation, the proteins were transferred to 0.2-μm Immun-Blot PVDF membranes (Bio-Rad). The membranes were blocked with 5% skim milk/TBS-T for 1 h at 20–25 °C, incubated for 12 h at 4 °C with primary antibodies, and incubated for 1 h with secondary antibodies. The blots were developed using the enhanced chemiluminescence reagent (Bio-Rad) and an automatic X-ray film processor (JPI Healthcare, Seoul, Korea). PPAR-γ was detected using the PPAR-γ antibody (Cell signaling Technology Inc., Danvers, MA, USA). The density of each band was normalized to that of GAPDH.

#### 2.5.3. Reporter Gene Assay for Evaluating TLR4 Activation

HEK-Blue-hTLR4 cells were seeded onto 96-well plates at a density of 2 × 10^4^ cells/well and incubated for 24 h. Test substances were diluted to a final concentration with reconstituted HEK-Blue™ Detection and added to each well. LPS (10 ng/mL) was used as a positive control. The plates were incubated at 37 °C for 48 h and the optical density of the samples was measured at 620 nm using a microplate reader.

#### 2.5.4. Reporter Gene Assay for Evaluating TGF-β Pathway Activation

To examine TGF-β/Smad transcriptional activity, A549 cells were transfected with a luciferase reporter containing the Smad-binding element reporter kit (BPS, San Diego, CA, USA). Transient transfection of the reporter construct was performed using Lipofectamine (Invitrogen, Carlsbad, CA, USA) according to the manufacturer’s instructions. A549 cells were treated with test substances individually or mixed at EC05 (5% effective concentration) for 48 h. Cell lysates were prepared using the luciferase lysis buffer (Promega, Fitchburg, WI, USA). Luciferase activity was determined in the cell lysates using the Dual-Luciferase^®^ Reporter Assay System kit (Promega) according to the manufacturer’s instructions. Renilla luciferase activity from co-transfected pRL-TK plasmid vector was used as a control for transfection efficiency.

#### 2.5.5. Statistical Analysis

Data were analyzed using SigmaPlot version 8 (Jandel Science Software, San Rafael, CA, USA) and Excel (Microsoft, Redmond, WA, USA). Statistical analyses were performed using SPSS version 18.0 (SPSS, Chicago, IL, USA), and differences between groups were assessed using Duncan’s posthoc test following one-way analysis of variance. Statistical significance was determined at *p* < 0.01 or *p* < 0.05.

## 3. Results

### 3.1. MD Modeling of PPAR-γ

**Figure 2:**
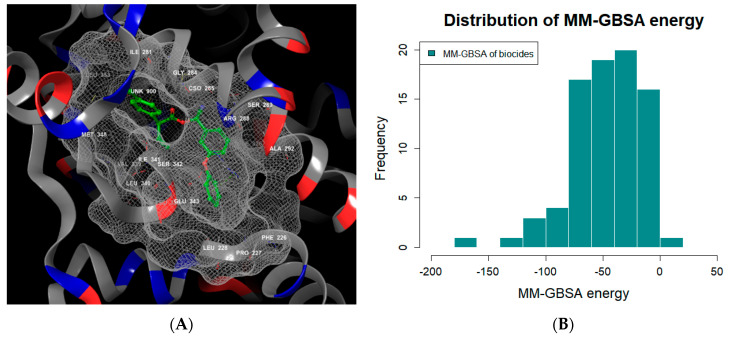
The result of molecular docking and MM-GBSA energy of molecular dynamics. (**A**) The biocide compound with a similar MM­GBSA score of GW9662 was docked onto inactive peroxisome proliferator-activated receptor-gamma (PPAR-γ) (PDB ID: 6C5T). This figure was generated using the Schrodinger Molecular Modeling Suite 2020-1. (**B**) The distribution of MM-GBSA energy values of 255 biocides is represented by the histogram.

MD were performed in 10 ns and the MM-GBSA values for 255 biocides were calculated (Figure 2). In order to set up the criteria of biocide that the compound has a binding affinity to inactive form of PPAR-γ, the MM-GBSA of four PPAR-γ reference antagonists was calculated using MD simulation. The docking score and MM-GBSA of PPAR-γ reference antagonists are listed in Table 2.

The reference antagonists of PPAR-γ are synthetic compounds for drug discovery. Because biocides have a different size than synthetic compounds, it is necessary to evaluate structural similarity compared with a biocide. Thus, similarity distances between biocides and four PPAR-γ reference antagonists were calculated based on Euclidean distance. The GW9662 reference antagonist was selected considering the criterion of similarity distance.

The prediction of a PPAR-γ inactivation with biocide was based on the criterion of the MM-GBSA of GW9662 (−69.71 kcal/mol). If the MM-GBSA of the biocide was lower than −69.71, it was predicted to a PPAR-γ inactivation compound. A total of 36 biocides were predicted to the compounds of PPAR-γ inactivation (Supplementary File S2).

### 3.2. Pharmacophore Modeling Results for TLR4

**Figure 3:**
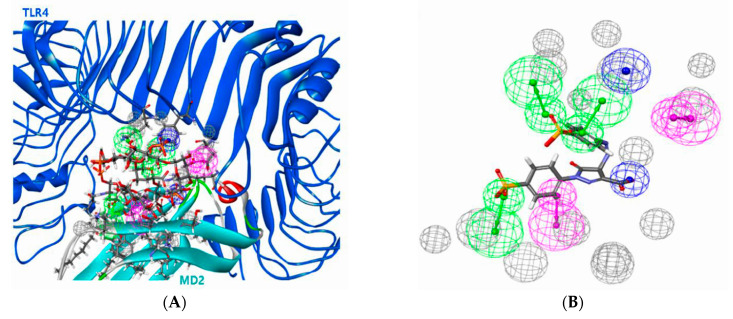
The 3D pharmacophore model. (**A**) The 3D pharmacophore model’s visualization generated considering TLR4, MD2, and whole LPS structural features in the TLR4-LPS-MD2 complex. (**B**) Pharmacophore features extracted from Lipopolysaccharides (LPS)-mimicking in the TLR4-MD2 complex. The mimic structure of the LPS was generated, considering the binding interaction between mimic structure and the protein complex. The model includes three hydrogen bond acceptors (green), two hydrogen bond donors (magenta), and two negative ionizable features (blue).

In order to screen the compound of TLR4 activation, a 3D pharmacophore model was generated from the crystal structure of human TLR4 with the LPS-bound MD-2 complex (Figure 3A). This model was built as an LPS-mimicking pharmacophore comprising chemical features required to form the TLR4-MD2 complex. The pharmacophore model comprises three hydrogen bond acceptors (green), two hydrogen bond donors (magenta), and two negative ionizable features (blue), excluding volume (gray) (Figure 3B).

In the pharmacophore model, the structure of the compound was mapped and the fit-value was calculated. The highest fit-value was obtained by mapping with five pharmacophoric features. A fit-value in the pharmacophore model indicates that the compound can bind to TLR4. Of the 255 biocides, fit-values were obtained for 25 compounds, and they were predicted to be potential compounds of TLR4 activation (Supplementary File S3). Chitosan (CAS No. 9012-76-4) compounds exhibited a high fit-value of 3.85. Prallethrin (CAS No. 23031-36-9) compounds exhibited a low fit-value of 0.19 in the pharmacophore model.

Compounds with a low fit-value of <1.0 accounted for 50% of the results. A compound including a fit-value of the pharmacophore model indicates that the compound is located at the TLR4 activation site. These compounds were considered TLR4 activation compounds.

### 3.3. QSAR Modeling Result

#### 3.3.1. Results of Data Curation

**Figure 4:**
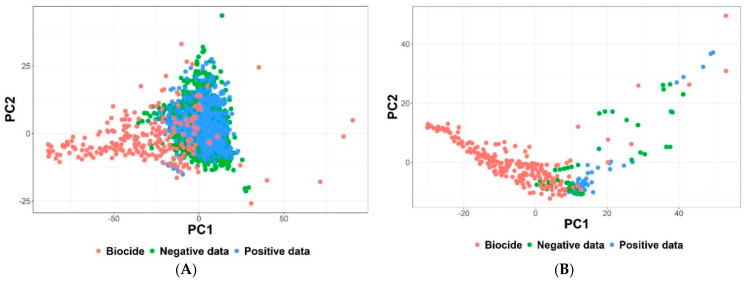
Plots of biocides versus peroxisome proliferator-activated receptor-gamma (PPAR-γ) inactivation and toll-like receptor 4 (TLR4) activation dataset. In the chemical space, biocides were compared with the (**A**) PPAR-γ dataset and (**B**) TLR4 dataset using physicochemical properties based on Euclidean distance.

To develop a reliable QSAR model for predicting whether a biocide is positive or negative in the MIE, we performed PCA and compared the collected dataset and biocides in the chemical space of physicochemical properties. Because there is a difference in the chemical space of physicochemical properties between collected dataset from PubChem and biocides, we filtered collected dataset in the chemical space using the average of 255 biocides similarity distance criteria (Figure 4).

A total of 3084 compounds for PPAR-γ inactivation and 118 compounds for TLR4 activation were filtered by the criteria of 1.24 and 1.22 average biocide similarity distance, respectively. If the collected data was below the average distance of 255 biocides, it was selected as modeling data. We selected 2784 compounds for PPAR-γ inactivation and 102 compounds for TLR4 activation as modeling dataset. For PPAR-γ inactivation, 813 compounds were positive dataset and 1971 compounds were negative dataset (ratio > 2:1). Because the PPAR-γ modeling dataset is unbalanced, we performed Tomek Link-based undersampling to fit a 1:1 ratio. Finally, 1626 modeling datasets of PPAR-γ, comprising positive dataset (813) and negative dataset (813), were selected (Supplementary File S4). Because the ratio of 102 compounds of TLR4 activation was close to 1:1, undersampling was not performed for the TLR4 modeling dataset. Finally, 102 modeling datasets of TLR4, comprising positive data (56) and negative data (46), were selected (Supplementary File S5).

#### 3.3.2. Classification-Based QSAR Models

Two classification-based QSAR models for PPAR-γ inactivation and TLR4 activation were developed using the SVM algorithm. The best descriptors of the QSAR models were selected based on model performance using the GA. The number of descriptors was 6 and 2 for PPAR-γ and TLR4, respectively (Table 3).

We developed a binary classification model using six descriptors to classify the compound as positive or negative of PPAR-γ inactivation. The molecular descriptors of the PPAR-γ QSAR model can be categorized into three types: (i) one with 2D atom-pairs features (B10[F-F], B10[S-CI]), (ii) the one with the functional group features (NCconj), and (iii) the one with topological indices features (MAXDN, SpMax7_Bh(e), SssCH2) associated with atoms’ electronegativity in the molecule. In the model, the descriptors of the topological indices that are associated with electronegativity showed the highest weight value. The descriptor with functional group features showed a second weight value. The lowest weight value was of descriptors with the 2D atom-pairs features. Among the three types of descriptors, electronegativity’s topological descriptors contributed to the compounds’ classification into positive or negative of PPAR-γ inactivation. Most of the negative compounds presented with features, such as descriptor MAXDN of 3–4, >7, descriptor SpMAX7_Bh(e) of <2, or descriptor SssCH2 of 15–20. In contrast, most positive compounds showed structural features that were in contrast to those of the negative compounds. The compound with PPAR-γ inactivation, such as 5-(3-bromophenyl)-1,3,4-oxadiazole-2-thiol (CAS No. 88466-20-0) or CHEMBL1299762 (CAS No. 29308-14-3), included atoms with strong electronegativity. These results showed the structural features related to atom with strong electronegativity may contribute to the prediction between positive and negative compounds of PPAR-γ inactivation.

In the binary classification model of TLR4 activation, we used two descriptors. The descriptor nR10 belongs to the ring descriptor, whereas F02[C-O] is included in the 2D atom pair. The F02[C-O] descriptor showed high weight in the SMV model, which was relatively more than that of the nR10 descriptor. In the model, compounds with the value of descriptor F02[C-O] of <2 or 15–20 and of descriptor nR10 of 2 showed TLR4 activation. Conversely, the negative compounds presented with features that were in contrast to those of the positive compounds. This result indicates that the positive or negative compounds of TLR4 activation can be classified according to the structural features.

The performance evaluation of each QSAR model was performed using average value of ACC, AUC, BA, MCC, sensitivity, and specificity of five external validation sets [40]. These performance indices are applied to evaluate the binary classification model. Additionally, the Y-randomization test of the QSAR models was performed to validate the robustness of the QSAR models (Supplementary File S6). The performance results obtained with the QSAR models are summarized in Table 4.

The average ACC and AUC of the two QSAR models of the internal and external validation dataset ranged from 0.82 to 1.00. In the performance result of external validation, two QSAR models showed the average ACC and AUC of >0.81. Moreover, the average sensitivity and specificity, and BA were observed between 0.81 and 0.96 among two QSAR models.

Although the MCC value was <0.70 in the QSAR model of PPAR-γ inactivation, other performance indices showed >0.80. Except for the QSAR model’s MCC value and because the overall performance indices of the two QSAR models showed >0.80, it implies that the two models exhibited good performance.

### 3.4. Application of MIE Modeling

#### 3.4.1. In Vitro Evaluation for MIE Regulation

The WST-1 cell viability assay was performed to determine the treatment concentration of the test substances. The maximum treatment concentration for MIE regulation was set at a level at which the cell viability decreased to ≤10.

**Figure 5:**
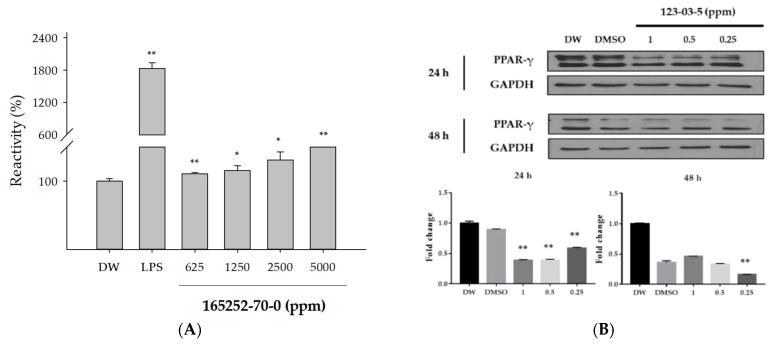
The effects of cetylpyridinium chloride (CAS No. 123-03-5) and dinotefuran (CAS No. 165252-70-0) on molecular initiating event (MIE) regulation. (**A**) HEK-Blue-hTLR4 cells were exposed to dinotefuran for 48 h. LPS (10 ng/mL) was used as the positive control. The data are expressed as percentage relative to the control (DW) group. * *p* < 0.05, ** *p* < 0.01 compared with the control (DW) group. (**B**) The expression of peroxisome proliferator-activated receptor-gamma (PPAR-γ) was evaluated using western blotting. DMSO was used as the vehicle control for cetylpyridinium chloride. The bar graph shows the quantified western blotting data. The data are expressed as fold-change relative to the control (DW) group. ** *p* < 0.01 compared with the vehicle control (DMSO) group.

A reporter gene assay was conducted to identify the compound of TLR4 activation. Significant TLR4 activation was shown in cells exposed to LPS, but not in most test substances predicted to compound of TLR4 activation. Only dinotefuran (CAS No. 165252-70-0) significantly increased the reactivity in HEK-Blue-hTLR4 cells exposed for 48 h (Figure 5A). Western blotting was performed to identify PPAR-γ inactivation. Similar to results for TLR4 activation, most test substances predicted to compound of PPAR-γ inactivation did not affect PPAR-γ expression. Only cetylpyridinium chloride (CAS No. 123-03-5) decreased PPAR-γ expression compared with vehicle control (DMSO) (Figure 5B). Because cetylpyridinium chloride was diluted with DMSO, A549 cells exposed to DMSO were used as the vehicle control.

#### 3.4.2. Comparison between MIE Modeling and In Vitro Testing

MIE modeling was used to predict whether the 255 biocides as compounds of PPAR-γ inactivation and of TLR4 activation. A total of 36 and 70 compounds of PPAR-γ inactivation were predicted using the MD and QSAR models, respectively (Supplementary Files S2 and S7). A total of 25 and 174 compounds of TLR4 activation were predicted for the pharmacophore and QSAR models, respectively (Supplementary Files S3 and S8). Of the compounds predicted using MIE modeling (100 for PPAR-γ inactivation and 191 for TLR4 activation), we selected compounds of PPAR-γ inactivation and TLR4 activation based on whether the compounds could be purchased and used for research. To evaluate the MIE modeling methods, we selected compounds of PPAR-γ inactivation and of TLR4 activation using different MIE modeling methods. In conclusion, we selected five compounds with PPAR-γ inactivation and five compounds with TLR4 activation and compared the ten predicted compounds using the MIE modeling methods and the in vitro results (Table 5).

Compared with in vitro results, MD exhibited an ACC of 0.20 in predicting compound of PPAR-γ inactivation. By contrast, the QSAR model exhibited an ACC of 0.80. The ACCs for predicting compound of TLR4 activation were 0.00 and 1.00 in the pharmacophore and QSAR models, respectively. In comparison with the MIE modeling, QSAR modeling exhibited high ACC than MD or pharmacophore modeling (Table 6). The accuracy of the QSAR method was >0.80, whereas other modeling methods showed values of <0.20. This result indicated that the QSAR method is relatively accurate compared with other methods in the MIE modeling of the two intersecting AOPs (AOP 347).

#### 3.4.3. In Vitro Validation of Selected Substances in the AOP Network

In the two intersecting AOPs (AOP 347), TLR4 activation and PPAR-γ inactivation were suggested as the MIE of pulmonary fibrosis. Both MIEs converged on the KE of “TGF-β pathway activation.” We attempted to confirm the application of MIE modeling by verifying whether each substance was linked to the following KE and whether the toxicity of the binary biocidal mixture was induced in converged KE. The reporter gene assay was performed to evaluate TGF-β/Smad transcriptional activity. A549 cells were treated with cetylpyridinium chloride and dinotefuran individually or mixed at EC05.

**Figure 6:**
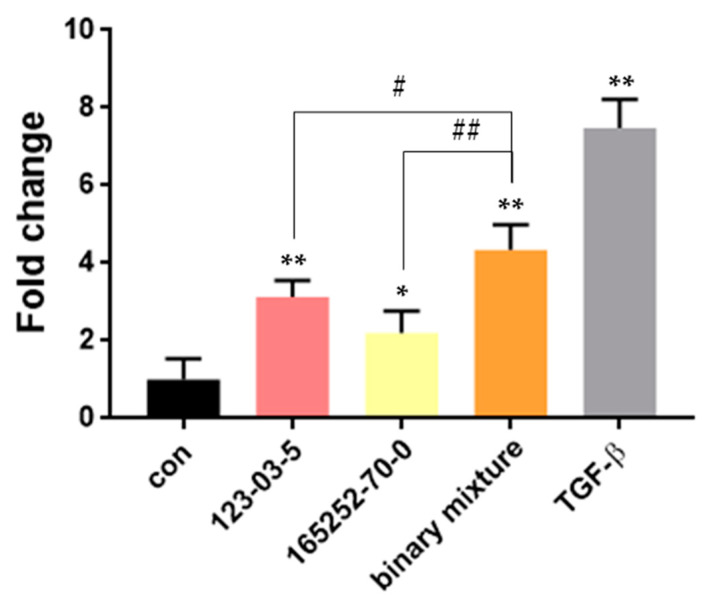
Effect of cetylpyridinium chloride (CAS No. 123-03-5) and dinotefuran (CAS No. 165252-70-0) on TGF-β pathway activation. A549 cells transfected with Smad-binding element luciferase reporter vector were treated with test substances either individually or mixed at EC05 (cetylpyridinium chloride: 0.0005 ppm; dinotefuran: 140 ppm) for 48 h. TGF-β (4 ng/mL) was used as the positive control. The data are expressed as fold-change relative to the control group. * *p* < 0.05, ** *p* < 0.01 compared with the control group. # *p* < 0.05, ## *p* < 0.01 compared with the single exposure group.

Each test substance significantly induced the TGF-β/Smad transcriptional activity, which was indicates that both MIE regulators induced the activation of the following KE. In addition, the binary biocidal mixture triggered a significant increase in TGF-β/Smad transcriptional activity compared with the single substance in the exposure group (Figure 6).

## 4. Discussion

In MIEs of the two intersecting AOPs (AOP 347), in vitro experiments were performed using five compounds of PPAR-γ inactivation and five compounds with TLR4 activation. Next, the in vitro experiment results were compared with the MIE modeling results. Although the number of positive compounds of each of the MIE was only one and the data were imbalanced, the MD and pharmacophore’s prediction ACC was noted to be relatively lower than QSAR modeling. Generally, the MD and pharmacophore methods are widely used in drug discovery and focused on synthetic compounds associated with drug discovery. In contrast to synthetic compounds, most biocide compounds are small in size, such as benzyl alcohol (CAS No. 100-51-6) and ziram (CAS No. 137-30-4). The physicochemical properties of biocides do not overlap with synthetic compounds significantly. Moreover, because the receptor of TLR4 comprises a complex structure with MD2 and has a wide binding pocket, the interaction of small compound is difficult for protein residues in the binding pocket. In the MD and pharmacophore modeling results, most of the biocides did not show MM-GBSA and fit-value. It means that the biocides do not fit into the MD and pharmacophore model. Therefore, the biocide prediction can be unsuitable for energy-based MIE modeling in the AOP 347 and may be inaccurate.

Conversely, the QSAR models were developed using a training dataset associated with biocides’ physicochemical properties. Because QSAR modeling employs features of molecular structures, the compound’s size does not significantly affect the prediction. However, if the QSAR model’s training dataset is dissimilar to biocides, the QSAR model may have poor prediction performance in biocide prediction. In MIE modeling of the AOP 347, QSAR modeling exhibited much better ACC than MD and pharmacophore modeling, indicating that the QSAR modeling can predict compounds small in sizes such as biocide better than MD or pharmacophore model.

From the results of both in silico and in vitro testing, we carefully suggest cetylpyridinium chloride (CAS No. 123-03-5) and dinotefuran (CAS No. 165252-70-0) as a potential binary combination of pulmonary fibrosis. According to a recent study, cetylpyridinium chloride showed a potential to induce apoptosis in human lung cells [41]. However, this binary combination for further experimental studies was not included in a real combined exposure scenario database for household chemical and biocidal products developed in our previous meta-study results [20]. We demonstrated that substances regulating MIEs activated the following KE, according to the AOP. In addition, the binary biocidal mixture significantly increased the activation of converged KE compared with that of a single substance. The interactions (including antagonism, potentiation, and synergies) of the binary biocidal mixture are unclear. However, these results indicate that the combination of in silico modeling and in vitro testing can efficiently screen for biocides that have the potential to reach AO. Although we suggested the MIE modeling as a QSAR method in the AOP 347, in vitro test data was not balanced because we selected positively predicted compounds using all MIE modeling methods. Therefore, we could restrictively evaluate MIE modeling methods in the AOP 347. In other AOP cases, applying the MIE modeling method to biocide research should be considered as the biocide and MIE receptor property, with the selection of a suitable MIE modeling method.

Global chemical regulations are continuously enhancing the chemical risk assessment framework; therefore, mixture toxicity can also be considered mainly in the risk assessment of mixtures. In the EU, the risk assessment of mixtures can be required generally in the following two regulatory frameworks: (i) substance- and product-based regulations for intentional mixtures such as the Registration, Evaluation, Authorization, and Restriction of Chemicals regulation (EC No. 2006/1907), the Placing of Plant Protection Products Regulation (EC No. 1107/2009), the BPR (EU No. 528/2012), and the Classification, Labeling, and Packaging for Dangerous Substances and Mixtures Regulation (EC No. 1272/2008); and (ii) process- and media-based regulations for coincidental mixtures such as the Water Framework Directive (Directive 2000/60/EC) and the Integrated Pollution and Prevention Control Directive (IPPC, Directive 2008/1/EC). In South Korea, Household Chemical Products and Biocides Safety Act (Chemical Product Safety Act, also known as K-BPR, Act No. 15511), considering the combined toxicity, entered into force in 2019 to strengthen the risk management of biocidal products. Nevertheless, the technical guidance documents for the risk assessment of mixtures to rapidly and sufficiently evaluate the combined toxicity under those regulations are still lacking. Thus, integrating in silico and in vitro based alternative methods (e.g., AOP framework) for use in animal tests has been increasingly developed and are highly expected to screen potential combined toxicities among mixture components [42].

## 5. Conclusions

In this study, biocides associated with pulmonary fibrosis were predicted based on the two intersecting AOPs (AOP 347) using MIE modeling methods. We selected five compounds of PPAR-γ inactivation and five compounds with TLR4 activation from the predicted compounds and performed in vitro experiments. Based on in vitro experiments, we compared the MIE modeling’s accuracy performance among MD, pharmacophore, and QSAR modeling. In the AOP 347, we showed that the QSAR model is a more competent method to predict biocide prediction than the MD and pharmacophore modeling. The two QSAR models were developed to screen biocidal active substances for the two MIEs in AOP 347; hence, the models can be employed to predict potential binary combinations that provoke pulmonary fibrosis owing to their joint effects. We suggest the novel QSAR models to the MIE modeling method in the AOP 347. Two QSAR models based on the AOP 347 would be a method for predicting chemicals of biocidal mixture associated with pulmonary fibrosis.

In addition, based on the MIE modeling method and in vitro testing, we finally proposed cetylpyridinium chloride (CAS No. 123-03-5) and dinotefuran (CAS No. 165252-30-0), as a potential binary biocidal combination of pulmonary fibrosis. Further studies on additional experimental tests need to be conducted to identify its mixture toxicity and validate the AOP 347.

## Figures and Tables

**Table 1:** Test substances predicted to be peroxisome proliferator-activated receptor-gamma (PPAR-γ) antagonists and toll-like receptor 4 (TLR4) agonists.

MIE ^1^	Substances	CAS No.	Prediction
PPAR-γ inactivation	Cetylpyridinium chloride	123-03-5	Positive (Antagonist)
	Cyfluthrin	68359-37-5	Positive (Antagonist)
	Deltamethrin	52918-63-5	Positive (Antagonist)
	Muscalure	27519-02-4	Positive (Antagonist)
	Piperonyl butoxide	51-03-6	Positive (Antagonist)
TLR4 activation	Propetamphos	31218-83-4	Positive (Agonist)
	Prallethrin	23031-36-9	Positive (Agonist)
	Novaluron	116714-46-6	Positive (Agonist)
	Imiprothrin	72963-72-5	Positive (Agonist)
	Dinotefuran	165252-70-0	Positive (Agonist)

^1^ MIE; Molecular initiating event.

**Table 2:** Docking and molecular dynamics (MD) results of reference antagonists.

Number	Reference Antagonists	CAS No.	Dock Score ^1^ (kcal/mol)	MM-GBSA ^2^ Score (kcal/mol)	Avg. Similarity Distance with Biocides ^3^
1	Betulinic acid	472-15-1	−6.36	−134.75	1.75
2	SR 1664	1338259-05-4	−11.90	−58.20	1.53
3	GW9662	22978-25-2	−7.66	−69.71	1.11
4	FH535	108409-83-2	−6.50	−50.70	1.38

^1^ The docking results were calculated using the Glide docking algorithm. ^2^ Molecular Mechanics/Generalized Born Surface Area. ^3^ Similarity distance between reference antagonists and biocides was calculated using Euclidean distance based on the molecular descriptors of alvaDesc.

**Table 3:** Selected molecular descriptors of the quantitative structure–activity relationship (QSAR) models based on alvaDesc.

QSAR Models	Descriptors	Description
PPAR-γ inactivation	B10[F-F]	Presence/absence of F-F at topological distance 10
	B10[S-Cl]	Presence/absence of S-Cl at topological distance 10
	MAXDN	Maximal electrotopological negative variation
	NCconj	Number of non-aromatic conjugated C (sp2)
	SpMax7_Bh(e)	Largest eigenvalue n. 7 OF Burden matrix weighted by Sanderson electronegativity
	SssCH2	Sum of ssCH2 E-state
TLR4 activation	nR10	Number of 10-membered rings
	F02[C-O]	Frequency of C-O at topological distance 2

**Table 4:** Performance indices of the quantitative structure–activity relationship (QSAR) models ^1^.

QSAR Models	Internal Validation (80% of Data Set)						External Validation (20% of Data Set)					
	ACC ^2^	AUC ^3^	MCC ^4^	Sensitivty ^5^	Specificity ^6^	BA ^7^	ACC	AUC	MCC	Sensitivity	Specificity	BA
PPAR-γ inactivation	0.82	0.87	0.63	0.83	0.80	0.81	0.82	0.88	0.64	0.83	0.81	0.82
TLR4 activation	0.98	1.00	0.97	0.99	0.97	0.98	0.97	1.00	0.97	0.96	0.94	0.95

^1^ Performance of the QSAR model is the average performance of five internal and external validation data sets. ^2^ ACC or accuracy is the ratio of the total number of correct predictions. ^3^ AUC; area under the curve. ^4^ MCC; Matthews correlation coefficient is used to evaluate binary classification performance. ^5^ Sensitivity is the proportion of positive class that is correctly identified. ^6^ Specificity is the proportion of negative class that is correctly identified. ^7^ Balance accuracy is the average of sensitivity and specificity.

**Table 5:** Comparison of in vitro experiments and molecular initiating event (MIE) modeling ^1^.

MIE ^2^	Substance	CAS No.	In Vitro	MD ^3^/Pharmacophore	QSAR ^4^
PPAR-γ inactivation	Cetylpyridinium chloride	123-03-5	Positive	Positive	Negative
	Cyfluthrin	68359-37-5	Negative	Positive	Negative
	Deltamethrin	52918-63-5	Negative	Positive	Negative
	Muscalure	27519-02-4	Negative	Positive	Negative
	Piperonyl butoxide	51-03-6	Negative	Positive	Negative
TLR4 activation	Propetamphos	31218-83-4	Negative	Positive	Negative
	Prallethrin	23031-36-9	Negative	Positive	Negative
	Novaluron	116714-46-6	Negative	Positive	Negative
	Imiprothrin	72963-72-5	Negative	Positive	Negative
	Dinotefuran	165252-70-0	Positive	Negative	Positive

^1^ Comparison between in vitro experiments and MIE modeling. ^2^ MIE; molecular initiating event. ^3^ MD; molecular dynamics. ^4^ QSAR; quantitative structure–activity relationship.

**Table 6:** Molecular initiating event (MIE) modeling performance results ^1^.

Performance Indices	PPAR-γ Inactivation		TLR4 Activation	
	MD ^2^	QSAR ^3^	Pharmacophore	QSAR
Accuracy	0.20	0.80	0.00	1.00

^1^ The performance results of each of the MIE modeling methods compared with in vitro experiments. ^2^ MD; molecular dynamics. ^3^ QSAR; quantitative structure–activity relationship.

## Data Availability

The data presented in this study are available in supplementary material here.

## Supplementary Materials

The following are available online at https://www.mdpi.com/2305-6304/9/3/59/s1, Supplementary File S1: 255 biocide compounds, Supplementary File S2: List of predicted PPAR-γ antagonists using MD, Supplementary File S3: List of predicted TLR4 agonists using a pharmacophore model, Supplementary File S4: QSAR modeling dataset of PPAR-γ, Supplementary File S5: QSAR modeling dataset of TLR4, Supplementary File S6: Y-randomization results of the QSAR models, Supplementary File S7: List of predicted PPAR-γ antagonists using the QSAR model, Supplementary File S8: List of predicted TLR4 agonists using QSAR model.
